# Cloning and Embryo Splitting in Mammalians: Brief History, Methods, and Achievements

**DOI:** 10.1155/2021/2347506

**Published:** 2021-11-30

**Authors:** Mohaddeseh Rahbaran, Ehsan Razeghian, Marwah Suliman Maashi, Abduladheem Turki Jalil, Gunawan Widjaja, Lakshmi Thangavelu, Mariya Yurievna Kuznetsova, Pourya Nasirmoghadas, Farid Heidari, Faroogh Marofi, Mostafa Jarahian

**Affiliations:** ^1^Animal Biotechnology Department, National Institute of Genetic Engineering and Biotechnology (NIGEB), Tehran, Iran; ^2^Human Genetics Division, Medical Biotechnology Department, National Institute of Genetics Engineering and Biotechnology (NIGEB), Tehran, Iran; ^3^Stem Cells and Regenerative Medicine Unit at King Fahad Medical Research Centre, Jeddah, Saudi Arabia; ^4^Faculty of Biology and Ecology, Yanka Kupala State University of Grodno, Grodno, Belarus; ^5^Universitas Krisnadwipayana, Jakarta, Indonesia; ^6^Department of Pharmacology, Saveetha Dental College and Hospital, Saveetha Institute of Medical and Technical Sciences, Saveetha University, Chennai, India; ^7^Sechenov First Moscow State Medical University, Moscow, Russia; ^8^Department of Medical Biotechnology, Faculty of Medical Sciences, Tarbiat Modares University, Tehran, Iran; ^9^Department of Hematology, Faculty of Medicine, Tabriz University of Medical Sciences, Tabriz, Iran; ^10^German Cancer Research Center, Toxicology and Chemotherapy Unit (G401), 69120 Heidelberg, Germany

## Abstract

Embryo splitting is one of the newest developed methods in reproductive biotechnology. In this method, after splitting embryos in 2-, 4-, and even 8-cell stages, every single blastomere can be developed separately, but the embryos are genetically identical. Embryo splitting, as an approach in reproductive cloning, is extensively employed in reproductive medicine studies, such as investigating human diseases, treating sterility, embryo donation, and gene therapy. In the present study, cloning in mammalians and cloning approaches are briefly reviewed. In addition, embryo splitting and the methods commonly used in embryo splitting and recent achievements in this field, as well as the applications of embryo splitting into livestock species, primate animals, and humans, are outlined. Finally, a perspective of embryo splitting is provided as the conclusion.

## 1. Introduction

In biology, cloning includes the reproduction of organisms without sexual intercourse. As a result, unlike sexual reproduction, progenies are not only collections of the characteristics of their parents but also the homological copies originating from the primary organism. In other words, cloning means biological materials, such as a gene, a cell, and even an organism for producing genetically identical copies [1]. These copies can be produced in vitro in two main ways, namely, somatic cell nuclear transfer (SCNT) and embryo splitting or twinning.

Embryo splitting is the in vitro stimulation of the natural process of producing identical twins with artificial SCNT [2]. For the first time, Solter and Magrath (1983) developed nuclear transfer technology in the mammalian embryo. In their study, a nucleus from a fertilized ovule was merged into another fertilized and nuclearized ovule. Next, the novel two-nuclear cell was transferred into the uterus of a female mouse [3]. After several experiments on mice at the Wistar Institute, Philadelphia, Solter (1984) stated that mammalian cloning is biologically possible.

Moreover, Willesden (1984), at the University of Cambridge, UK, cloned a sheep utilizing embryonic stem cells (ESCs) [4], as well as attempting to create sheep-goat and sheep-cow hybrids by merging embryonic cells (ECs) of several mammalian species. Willesden (1986) merged denuclearized ovules of sheep with blastomeres split from eight-cell-stage embryos to produce novel sheep [5]. Sims and First utilized cow ESCs for cloning with a cow embryo first developed in vitro in 28 days. Afterwards, the cells were split and the nucleus was removed. Approximately 24% of zygotes reached the blastocyst stage, and 12% of the blastocysts were transferred into the uterus of the cattle for embryo growth and development [6]. It has been demonstrated that the nucleus separated from the two-cell-stage embryo can directly reach the blastocyst stage.

In contrast, the embryo will not be able to grow if the nucleus is separated from other embryonic stages. Furthermore, many reports indicated that blastomeres are still totipotent in the primary stage. In other words, they are still able to reach a full organism [7]. Finally, Wilmoth (July 1996) succeeded in creating the first cloned organism, a sheep known as Dolly, utilizing mature cells from the mammary gland of a sheep [3]. The present review has focused on the main cloning approaches in mammalians with an emphasis on embryo splitting.

## 2. Cloning Approaches

Various approaches have been found for cloning that is specific to the types of organisms, such as animals, plants, fungi, and bacteria. However, three approaches of molecular, cellular, and organismal cloning are widely considered and used in animals, especially mammalians [4].

### 2.1. Molecular Cloning

**Figure 1:**
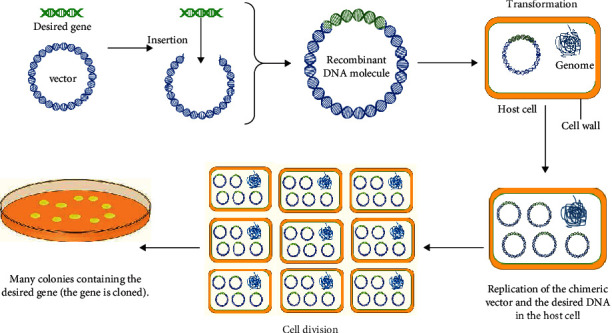
Molecular cloning process.

Molecular cloning, also known as DNA or gene cloning, was developed during 1971-1973 when genetic engineering was introduced. The technique is effective for producing many homologous copies from a DNA fragment. First, a DNA fragment, containing the desired gene, is cleaved, then is inserted into a circular DNA molecule as a vector for producing chimeric or recombinant DNA molecules [8, 9]. A vector is a transporter tool for transferring the desired gene into the host cell, such as a virus and bacterium. Following transferring a vector into a host cell, this vector is replicated to produce a variety of homologous copies of the vector and consequently the desired gene. Moreover, during cell division, the novel vectors can be transferred to the offspring. As the result of consecutive vector replications and cell division, a colony is created from many identical copies containing numerous copies of the recombinant DNA molecule. Therefore, the desired gene, inserted into the vector, can be replicated many times; in biological terms, the gene is cloned (Figure 1) [10].

The technique has been beneficial for achieving significant success in the treatment of human diseases and production of vital recombinant medications, such as insulin for diabetic patients, tissue plasminogen activator for the elimination of blood clots in heart attack, and erythropoietin for the anemia of the dialysis patients [11]. However, some problems may be encountered in gene cloning. For example, large DNA fragments cannot be inserted into the vectors, and some genes, particularly those which are derived from eukaryotes, are too large. They might be destructed during vector recombination. As the second problem, the sequences of numerous genes are yet unknown. Consequently, it could be difficult to find the accurate desired DNA fragment to clone and produce a recombinant protein [9]. The last concern is gene escape, which means that a cloned or a selectable marker gene might be transferred into wild organisms. Gene escape can promote a trait in the acceptor organisms. As an example, antibiotic resistance genes are widely used as selective marker genes. If the genes are unintentionally transferred into an infectious bacterium, the organism will resist a specific antibiotic [8].

### 2.2. Cellular Cloning

Cellular cloning is used on a cellular scale to create vast homologous copies of a specific cell by splitting a single cell from an organism *in vitro*. In other words, cell cloning is the process of expanding a cell to a cell population. The process is highly simple in single-cell microorganisms, such as bacteria and yeasts, and just needs incubation in a proper medium. On the other hand, multiple-cell organism culturing is more difficult, because the cells derived from these organisms cannot easily grow on the media [11]. It is noteworthy that sexual cells, such as sperm and ovule, do not play any roles in molecular and cellular cloning. The cloned cells are not susceptible to developing a full multicellular organism, such as an animal or a plant [12].

The purified cloned cells are known as a cell line that is genetically similar to the first cell. The technique is valid for producing cell lines to be used in cellular and pharmaceutical research [12]. Cloning rings (cylinders) could be used for generating a purified cell line from a tissue. In this method, a suspension is provided from the single cells of the desired tissue and is then treated by a mutagen or a specific medication to select the cells with a specific characteristic [12]. Moreover, a diluted suspension can be cultured in a proper solid medium to produce a single-separated colony of single cells. The cell suspension should be diluted to the extent that the cells are separately located on the medium and a colony is derived from the replication of each of them. In the primary stages, a sterilized (cloning) ring is located around each growing colony and a little trypsin is added to the ring. Finally, each colony is transferred into a segregated medium [12].

### 2.3. Organismal Cloning

Unlike molecular and cellular cloning, organismal cloning, also known as reproductive cloning, can produce genetically identical copies for creating a full multicellular organism, such as a plant, an animal, and even a human. Reproductive cloning could be categorized as natural twinning, therapeutic cloning, SCNT, and embryo splitting [13], which are discussed here.

#### 2.3.1. Natural Twinning

**Figure 2:**
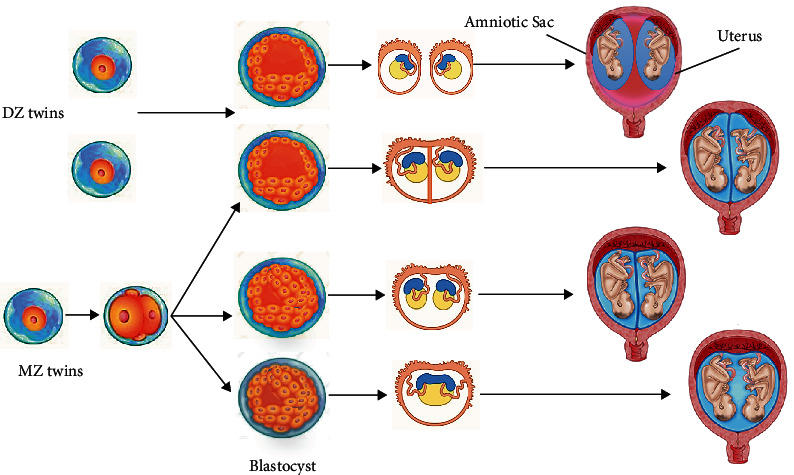
Natural twinning process.

Two types of twinning can be found in nature, namely, monozygotic (MZ) or identical twins observed in 3 cases per 2000 parturitions and dizygotic (DZ) or fraternal twins reported to be approximately twice MZ twins [14]. Identical twins are physically similar and also genetically homologous with the same genomic sequences because they originate from one zygote. Therefore, two homologous cells are created by the first zygote division. The cells separately divide and develop to generate two full organisms as two examples of the miniclone (Figure 2) [15] causing the gender of identical twins to be the same. On the other hand, DZ twins result from the fertilization of two ovules by two sperms; consequently, they can be sexually and genetically different. The resemblance of their genetic content is as similar as ordinary siblings, and DZ twins are not considered as a clone [16].

#### 2.3.2. Therapeutic Cloning

**Figure 3:**
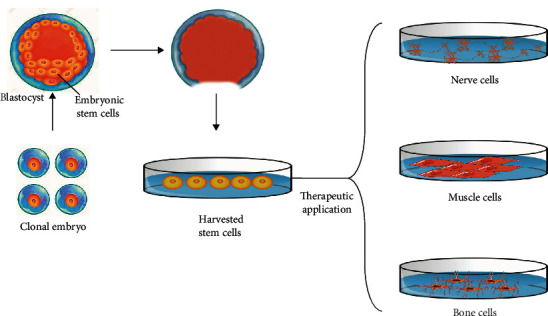
Isolation of stem cells from a preembryo.

Utilized techniques in therapeutic or biomedical cloning, known as research cloning, exactly resemble the methods used in reproductive cloning, particularly SCNT, whilst the formed preembryo is not transferred into the uterus. The preembryos are applied for isolating ESCs after 4-5 days of formation (Figure 3). The ESCs are isolated to produce tissues and organs for implantation. Overall, the main goal of therapeutic cloning could be the in vitro regeneration of tissues and organs [17].

#### 2.3.3. SCNT

**Figure 4:**
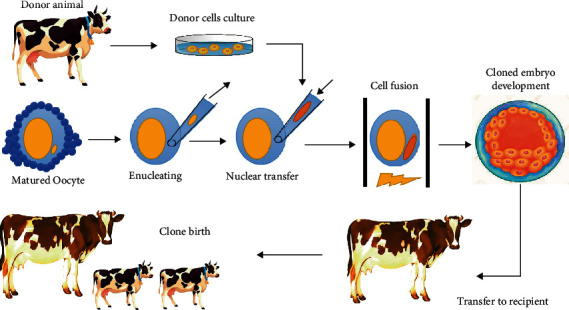
SCNT process steps.

In SCNT or adult DNA cloning, as an approach of organismal cloning, the oocyte nucleus is removed, and the nuclear genetic content of the somatic cell is inserted into the denuclearized oocyte. After fertilizing the novel oocyte, this new preembryo is transferred into the host uterus for creating a full organism following the implantation and development of the novel embryo [18]. The first stage of SCNT, known as nucleation, is the elimination of the haploid chromosomes (n) that contain the meiotic spindle complex of the oocyte in metaphase stage II. Nucleation is followed by the transportation and fusion of diploid somatic cells (2n) derived from a proper donor into an oocyte without nuclear. The final cell is known as a cytoplast [19] and is artificially activated by electric pulses or chemical stimulation for further embryo development (Figure 4) [18]. It should be noted that this approach was employed for creating Dolly and other cloned organisms. Moreover, SCNT was used for successfully cloning other species, such as cattle, mice, sheep, pigs, rabbits, and rhesus monkeys to generate ESC and live offspring for reproductive/therapeutic cloning. Since 20 years ago, SNTC has been used in stem cell research and regenerative medicine [3].

Although SCNT is theoretically easy to use, there are many problems in practice that reduce the efficiency [18]. The SCNT with any type of donor cell can be fatal before, after, and during the nesting stage and all the stages of growth pre- and postparturition. The first phenotype and defect of cloning are stoppage in cell division and genome instability that even happen before transcriptional abnormalities. It could indicate that epigenetic processes involved in diverse situations can affect not only transcription but also DNA replication. Reprogramming is restricted by, first, genome instability and, second, transcriptional defects; the first is followed by the second one [20]. The rate of abortion and perinatal mortality caused by growth defects in the cloned live offspring of several species is high. Abortion and perinatal mortality are related to the incomplete reprogramming of somatic nuclei by SCNT [19]. Inadequate nuclear reconstructing and reprogramming may cause abnormal gene expression leading to the abnormal placenta and fetus growth. Large offspring syndrome is the next negative consequence of using SCNT. During pregnancy, some phenotypes can be observed, such as hydroallantois, decreased breast growth, and long-term pregnancy [20]. Other negative at-birth phenotypes include overweight, abnormal limb size, lost motion control, tongue enlargement, respiratory problems, and vulnerable immune system [19]. There are several technical factors, including invasive microscopic manipulation, oocyte inability, changes in growth efficiency, and incompatible *in vitro* culture [18].

### 2.4. Embryo Splitting

Embryo splitting or embryo twinning refers to the formation of twins or multiple embryos in vitro to split an embryo in 2-, 4-, or 8-cell stages. The blastomeres can be still totipotent at the initial stage of embryogenesis. The ability has been considered for the in vitro production of a full organism, as well as utilizing ESCs in biomedical cloning [21]. In many studies, it has been reported that splitting the 6- to the 8-cell embryo can be developmentally more efficient than the 2- to 5-cell-stage embryos. Embryo splitting could be beneficial in providing further embryos for patients who are least stimulated by hormone therapy in reproduction programs [22]. Embryo splitting is the same as the natural process of creating identical twins. Numerous advantages have been found for embryo splitting in research and reproduction programs. First, when the ovary contains a low number of oocytes and the chance of embryo formation is considerably poor, embryo splitting can be used to provide sufficient embryos for transferring one of them into the uterus. The other embryos can be frozen for later implantation [2]. As the second merit, genetic diseases could be diagnosed before the implantation of the formed embryo. For this purpose, an embryo is split to create a twin, one of which is used for diagnosis and the other is cultured to create a full organism. The third advantage is the treatment of genetic diseases by gene therapy at the earliest stage of embryo formation [23]. Furthermore, the fourth benefit of an embryo splitting is the in vitro production of tissues or organs. In other words, if the offspring needs tissue or organ transplant, the other embryo, protected in the reproductive biological laboratory, can be used to produce the tissue or organ [3].

#### 2.4.1. Methods of Embryo Splitting

Based on the embryogenesis stage, the techniques, employed for embryo splitting, can be either blastomere biopsy or bisection for cleavage-stage embryo and morula or blastocyst, respectively. Generally, there is no report to show that biopsy and bisection significantly influence the rates of twinborn or twin pregnancy [23]. These techniques are briefly explained in the following sections.

**Figure 5:**
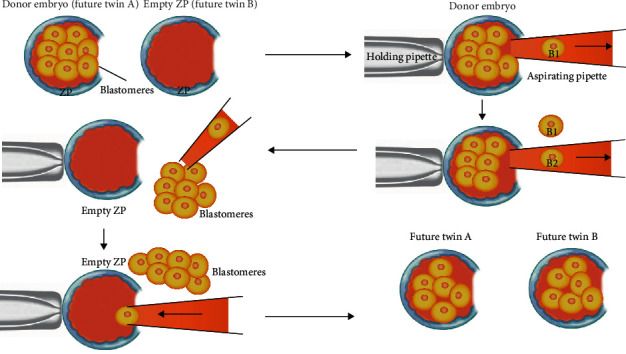
Blastomere biopsy/separation stages. B: blastomere.

*(1) Blastomere Biopsy/Separation*. Separating blastomere includes removing one or more blastomeres and inserting them into a prepared and evacuated zona pellucida (ZP) for growth and development. Initially, the embryo donor is treated with Tyrode's solution to open a hole in ZP. Next, blastomeres are removed from ZP by an aspirating pipette through the hole. These free blastomeres are transferred into the empty ZP (Figure 5) [22]. The technique has been successfully tested in large animal species, particularly farm animals, including sheep, cattle, horses, and pigs, but not in nonhuman primates especially *rhesus* monkeys [23].

**Figure 6:**
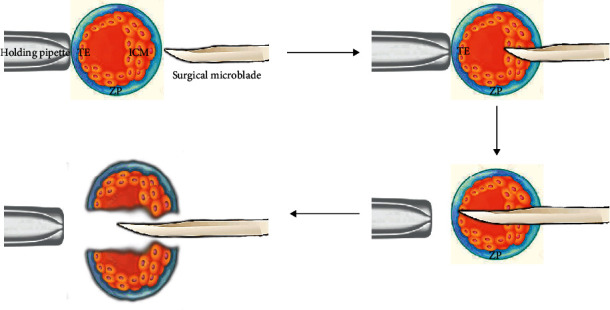
Blastocyst bisection stages.

*(2) Bisection*. This method is employed for mechanically dividing a compact embryo into two parts that equally contain blastocysts, trophectoderm, and inner cell mass (Figure 6). The embryos of MZ twins can be split by this method, then immediately cultured in a medium for further growth and development [24]. It has been repeatedly reported that the bisection of blastocysts is effective for large mammalian species, specifically livestock, such as sheep, cattle, goats, and pigs. However, the technique has not yet been tested in *Homo sapiens* [23]. Blastomere biopsy might be accompanied by various advantages and disadvantages, compared to bisection (Table 1).

#### 2.4.2. Embryo Splitting in Livestock

Many studies reported the high efficiency of embryo splitting in farm animals. In sheep, for instance, 36% of split embryos in the 2-4-cell stage developed to full organisms following transfer into the uterus. In addition, MZ multiple calves, derived from splitting cattle embryos, were successfully and healthily born [25]. The split embryos of a pig can completely grow to develop piglet twins. Moreover, horse embryos, split by 2- to 8-cell-stage blastomere biopsy, can create healthy full MZ offspring [26]. There is a brief report of using embryo splitting in livestock science in Table 2.

#### 2.4.3. Embryo Splitting in Nonhuman Primates

Before embryo splitting in humans, the technique was first tested in rhesus macaque (*Macaca mulatta*), a species of old world monkey, as a nonhuman primate model evolutionarily, genetically, and physiologically related to *H. sapiens* [27]. Therefore, the primates can be used for human research to gain key and vital information, particularly concerning the successful development of techniques to produce identical twins in primates which significantly promotes understanding diseases and MZ twinning in *H. sapiens*, as well as how the maternal environment affects the epigenetic profile of the human embryo [28]. Moreover, these studies can make primates more effective animal models for research in vaccination and the implantation of tissues [27]. Similar successful embryo twinning in farm animals has not been observed in rhesus monkeys due to diverse reasons. Monkey does not naturally carry twins, and only about 0.25% of all pregnancies are twins. In addition, the offspring may be sometimes faced with various complications leading to death [29]. The rate of the pregnancy after transferring two split embryos is estimated at 25%-40%. However, the rate of twin pregnancies is less than 15%. Transferring a single embryo derived from twin or multiple pregnancies into female individuals can significantly improve the results and efficiency [30].  Table 3 represents a summary of the studies on embryo splitting in the rhesus macaque.

#### 2.4.4. Embryo Splitting in Human

First, embryo splitting in a human was reported by a research group from George Washington University in Washington, DC, USA, at the joint meeting of the American Fertility Association and Canadian Fertility and Andrology Society in October 1993 [31]. In the latter study, the blastomeres were split from seventeen 2-8-cell embryos, covered in ZP, and cultured to reach the 32-cell stage. It has been claimed that the achievement can be beneficial in treating infertility in humans [32]. However, the investigation had not been confirmed by a valid institutional supervisory board. Therefore, the research team was reprimanded and instructed for removing the data. The case caused intense ethical debate about embryo cloning, followed up by the Ethics Committee of the American Society for Regenerative Medicine to publish a statement about using embryo splitting for infertility treatment [23]. A brief report of a human embryo splitting is available in Table 4.

## 3. Bioethics in Cloning

There are many controversial and serious concerns about the probable misemployments and unpredictable effects of human cloning. Proponents and opponents of human cloning provide their opinions on different aspects, including biology, medicine, sociology, philosophy, theology, economics, and politics [33]. Generally, the proponents mention the remedial benefits of human cloning, such as the treatment of infertility, neurological issues, cardiovascular diseases, diabetes, AIDS, and other immune system disorders. Generating and harvesting SCs can be considered as one of the most vital consequences of human cloning that are beneficial for tissue regeneration and organ implantation [34]. On the other hand, the opponents believe that human cloning is against human dignity. Furthermore, there is an ambiguity in the lineage of the cloned individuals, and the relation between the cloned individuals and their origins is not clear [33].

According to the ambiguities and concerns, and also the medical benefits of human cloning, international and regional organizations have legislated some rules not only to restrict the probable misemployments of human cloning but also to facilitate the medical application [35]. An optional protocol of the Council of Europe in the convention for the Protection of Human Rights and Dignity, 12 January 1998, states that any intervention to create a human genetically identical with a live or dead human is banned. In addition, article 9 of the protocol claimed that the exploitation of generic identical humans is contrary to human dignity [36]. The first article of the UNESCO declaration on the human genome declares that the human genome is the basis of the fundamental unity of all members of the human family, as well as the recognition of their inherent dignity and distinction. Any actions for human cloning that are contrary to human dignity will not be allowed. The declaration asked competent governments and international organizations to collaborate to take some essential measures in national and international scales for observing the principles [37]. Moreover, the World Health Organization has emphasized in two resolutions in 1997 and 1998 that human cloning is morally contrary to human dignity [33, 37].

## 4. Recent Achievements

### 4.1. Embryo Splitting

Over the last decade, embryo splitting has been molecularly and cellularly evaluated. Velasquez et al. studied the morphology of bovine embryo split through blastocyst bisection, as well as the expression of some genes, including *OCT4*, *SOX2*, *NANOG*, *CDX2*, *TP1*, *TKDP1*, *EOMES*, and *BAX*. They found that the morphological characteristics of the split embryos differed significantly after 13 days. Moreover, *OCT4*, *SOX2*, *TP1*, and EOMES expression was decreased by embryo splitting [38]. The miRNA profile was investigated in human embryos split using blastomere biopsy. As a result, six miRNAs were significantly abundant in these embryos, whilst 22.9% of miRNAs were not detected [39].

In addition, Velasquez et al. evaluated the influence of bovine embryo splitting on gene expression during the elongation stage by bioinformatic tools. They observed the expression of the genes involved in growth, detoxification, matrix remodeling, and metabolite transport [40]. Tu et al. utilized embryo splitting techniques for editing the genome of cynomolgus monkeys (*Macaca fascicularis*) as a nonhuman primate via CRISPR/Cas9 [41].

Omidi et al. attempted to generate human ESC using embryo splitting techniques; however, the quality of the generated SCs was poor [18]. They also studied the efficiency of human embryo splitting sources, including chromosomally abnormal embryos, parthenogenetic embryos, frozen-warmed donated embryos, and embryos derived from the fertilization of in vitro matured oocytes. The results indicated the highest efficiency of splitting into frozen-warmed embryos and chromosomally abnormal embryos [25]. Other recent studies in this regard are briefly represented in Table 5.

### 4.2. Mitochondrial Replacement Techniques (MRTs)

The MTRs entail a group of related embryological methods that can be employed to prevent transmitting a pathogenic gene of the mitochondrial DNA (mtDNA) from mother to offspring. Consequently, MTRs can avoid serving mtDNA-dependent mitochondrial diseases limiting and threatening life [42]. Maternal spindle transfer (MST) and pronuclear transfer (PNT) are considered the two most common versions of MRTs. The MST and PNT are performed before and after fertilization, respectively. During MST, the spindle that contains maternal chromosomes can be transferred from an arrested oocyte of the prospective mother in metaphase II to a spindle-removed donor oocyte [43]. During PNT, newly formed nuclei are transferred to a fertilized and nuclear-removed donor oocyte. In MST and PNT, the donor oocyte is derived from a woman without any genetic disorders. Therefore, the regenerated embryo has a replaced normal mtDNA with the lowest level of the probable maternal pathogenic mtDNA [44].

Generally, MRTs include the transmission of the zygotic and meiotic genomes into familiar cellular environments. The techniques do not make any specific risks related to the wide reprogramming of the nuclear genome of the differentiated adult cell. Very limited clinical and preclinical data indicate an acceptable efficiency to use controlled-clinical MTR for preventing the transmission of mitochondrial disorders in particular limited conditions under precise supervision [45]. This is an assisted reproductive technology allowing socially and scientifically new movement in a familiar reproductive atmosphere. MRT is a sexual reproduction, though it is mechanically unusual [43].

### 4.3. Regenerative Medicine and SCs

The SC research is generally a promising field for the remedy of many diseases, for which there is currently no treatment. Many attempts have been performed to discover novel methods, such as genetic reprogramming techniques, for generating SCs from other cells. Although no spontaneous and specific function has been observed for SCs, they are crucial for many reasons. As the most prominent one, they can be induced to differentiate to every type of specialized cell. Next, the new cells can be used to repair the damaged tissues. As a result, the replacement of lost and damaged cells could be a remedial application of SCs [46]. In the following parts, the most remarkable SC remedial applications are outlined:

#### 4.3.1. Tissue Regeneration

This can be regarded as the first application of SCs. Usually, patients who need a new kidney, heart, lung, or pancreas have to expect a proper donor for implantation. Because of the permanent shortage of organs for donation to patients in need of implantation, SC programming for differentiation can be employed to generate a specific tissue or organ. Recently, SCs from just below the surface of the skin are used to create new skin tissue. Tissue transplant can repair injuries, severe burns, and other kinds of damage leading to the growth of new skin cells [47].

#### 4.3.2. Treatment of Neurodegenerative Diseases

The diseases associated with degeneration of the neurons and other nerve cells are known as neurodegenerative diseases, including Alzheimer's disease, Parkinson's disease, and multiple sclerosis [48]. There have been many efforts to use SCs to repair and regenerate damaged brain and other nerve tissues. Many studies have reported ESCs as the most efficient tools for the cell therapy of neurodegenerative diseases [49].

#### 4.3.3. Treatment of Blood Diseases

Currently, many hematologic diseases, such as leukemia, sickle cell anemia, and immune deficiencies can be treated by hematopoietic SCs (HSCs). The HSCs are found in the blood and bone marrow being able to generate all types of blood cells, including red and white blood cells [50].

#### 4.3.4. Remedy for Age-Dependent Ocular Macular Degeneration

It can be the most crucial reason for blindness in the elderly. Visual perception can be often lost by the dysfunction of retinal pigment epithelial cells (RPECs) in some patients. The SCs could provide a remedial approach to treat the disorders. Induced pluripotent SCs can be employed for the *in vitro* generation of RPECs to replace damaged cells by surgery [51].

## 5. Conclusion

The embryo splitting technique has significantly developed in farm animals, particularly cattle. Therefore, this method will be used as an effective approach to animal cloning in animal breeding and biotechnology. It can be predicted that the chance of successful pregnancy and twinning will be enhanced in nonhuman primates. The MZ twins of rhesus monkeys could be employed to study twinning and tissue implantation in humans, as well as the influences of epigenetic factors derived from the maternal environment on embryogenesis. Blastomere biopsy could be used for creating human embryonic stem cell lines. Finally, embryo splitting can be utilized for therapeutic application in reproduction programs and, consequently, the legislation of new roles and laws concerning legal issues related to human embryo splitting.

## Figures and Tables

**Table 1:** Comparing blastomere biopsy and bisection.

	Growth stage of embryo	Advantages	Disadvantages	References
Blastomere biopsy	Cleavage stage	Applicable for preimplantation	Low efficiency in 8-cell stage in some species	[34]
Bisection	Morula and blastocyst	Creating MZ twinning in mice, cow, goat, & pig	Cell damage	[35]

**Table 2:** Studies in livestock embryo splitting.

Species	Embryo sample	Method	Efficiency	Reference
Sheep	Two- & four-cell	Biopsy	36%	[36]
Bovine	Eight-cell	Biopsy	18%	[37]
Bovine	Morula	Bisection	66.6%	[38]
Bovine	Morula	Bisection	62.5%	[39]
Pig	Morula	Bisection	25%	[40]
Goat	Morula	Bisection	37%	[41]
Horse	Two- & eight-cell	Biopsy	50%	[42]
Bovine	Morula	Bisection	63.2%	[43]
Bovine	Morula	Bisection	53%	[44]
Goat	Morula	Bisection	59%	[45]
Sheep	Morula	Bisection	68%	[46]
Pig	Morula	Bisection	30%	[47]
Bovine	Morula	Bisection	74.6%	[48]
Sheep	Eight-cell	Biopsy	23.2%	[49]
Bovine	Morula	Bisection	69.2%	[50]

**Table 3:** Experiments in rhesus macaque embryo splitting.

Embryo sample	Method	Efficiency	Reference
8–16-cell	Biopsy	31%	[51]
2- & 4-cell	Biopsy	33%	[52]
8-cell	Biopsy	22%	[53]
8-cell	Biopsy	34%	[54]
8–16-cell	Biopsy	26%	[55]
8-cell	Biopsy	29%	[56]
Blastocyst	Bisection	36%	[57]
8-cell	Biopsy	32%	[58]

**Table 4:** Human embryo splitting.

Embryo sample	Method	Results	Reference
4-cell	Biopsy	*In vitro* developing into blastocysts	[59]
4-cell	Biopsy	Harvesting human ESCs (hESCs)	[60]
2- to 5- & 6- to 8-cell	Biopsy	First twined human embryos	[28]
Morula	Bisection	Harvesting hESCs	[61]
4-cell	Biopsy	Harvesting hESCs	[62]
Blastocyst	Bisection	Harvesting hESCs	[63]
4-cell	Biopsy	First pregnancy by embryo splitting	[64]
4-cell	Biopsy	Harvesting first Swiss hESC (CH-ES1)	[65]
4-cell	Biopsy	Harvesting hESCs	[66]

**Table 5:** Recent studies in embryo splitting.

Species	Embryo sample	Method	Results	Reference
Human	4-cell	Biopsy	*NANOG* expression in TE & ICM	[59]
Mouse	2-cell	Biopsy	*Oct4* expression in blastocysts	[67]
Mouse	2-cell	Biopsy	*Sox2* expression in blastocysts	[68]
Mouse	2-cell	Biopsy	*Cdx2* expression in blastocysts	[69]
Mouse	2-cell	Biopsy	*NANOG* expression in blastocysts	[70]
Mouse	2-cell	Biopsy	100% *Arntl* and 91% *Prrt2* knockout by C-CRISPR	[65]
Cynomolgus monkeys				
Cynomolgus monkeys	2-cell	Biopsy	Dax1 knockout by CRISPR/Cas9	[71]
Rhesus monkey	4-cell	Biopsy	Dystrophin gene knockout by CRISPR/Cas9	[72]
