# Corrigendum to “Cloning and Embryo Splitting in Mammalians: Brief History, Methods, and Achievements”

**DOI:** 10.1155/2024/9837676

**Published:** 2024-07-17

**Authors:** Mohaddeseh Rahbaran, Ehsan Razeghian, Marwah Suliman Maashi, Abduladheem Turki Jalil, Gunawan Widjaja, Lakshmi Thangavelu, Mariya Yurievna Kuznetsova, Pourya Nasirmoghadas, Farid Heidari, Faroogh Marofi, Mostafa Jarahian

**Affiliations:** ^1^ Animal Biotechnology Department National Institute of Genetic Engineering and Biotechnology (NIGEB), Tehran, Iran; ^2^ Human Genetics Division Medical Biotechnology Department National Institute of Genetics Engineering and Biotechnology (NIGEB), Tehran, Iran; ^3^ Medical Laboratory Technology Department Faculty of Applied Medical Sciences King Abdulaziz University, Jeddah 21589, Saudi Arabia; ^4^ Faculty of Biology and Ecology Yanka Kupala State University of Grodno, Grodno, Belarus; ^5^ Universitas Krisnadwipayana, Jakarta, Indonesia; ^6^ Department of Pharmacology Saveetha Dental College and Hospital Saveetha Institute of Medical and Technical Sciences Saveetha University, Chennai, India; ^7^ Sechenov First Moscow State Medical University, Moscow, Russia; ^8^ Department of Medical Biotechnology Faculty of Medical Sciences Tarbiat Modares University, Tehran, Iran; ^9^ Department of Hematology Faculty of Medicine Tabriz University of Medical Sciences, Tabriz, Iran; ^10^ German Cancer Research Center Toxicology and Chemotherapy Unit (G401) 69120, Heidelberg, Germany

In the article titled “Cloning and Embryo Splitting in Mammalians: Brief History, Methods, and Achievements” [1], the incorrect affiliation was listed for Marwah Suliman Maashi and the authors have requested to correct the affiliation, as above.
